# Size matters: larger fragments of riparian forest in urban areas support functional diversity of soil bacteria more than smaller ones

**DOI:** 10.3389/fmicb.2025.1517545

**Published:** 2025-02-26

**Authors:** Gabriela Koster, Małgorzata Jaźwa, Sebastian Wojciech Przemieniecki, Łukasz Musielok, Hamed Azarbad, Beata Klimek

**Affiliations:** ^1^Faculty of Biology, Institute of Environmental Sciences, Jagiellonian University, Kraków, Poland; ^2^Faculty of Natural Sciences and Technology, Institute of Biology, University of Opole, Opole, Poland; ^3^Department of Entomology, Faculty of Agriculture and Forestry, Phytopathology and Molecular Diagnostics, University of Warmia and Mazury in Olsztyn, Olsztyn, Poland; ^4^Faculty of Geography and Spatial Management, Jagiellonian University, Kraków, Poland; ^5^Department of Biology, Evolutionary Ecology of Plants, Philipps-University Marburg, Marburg, Germany

**Keywords:** bacterial communities, functional diversity, microorganisms biogeography, riparian forests, urban soils

## Abstract

Soil microorganisms are relatively poorly studied in urban ecosystems, particularly within unmanaged woodlands that form island-like patches of vegetation. We surveyed soil bacteria on *Salix* spp. dominated riparian-like forest patches in Kraków, the second largest city in Poland, to find out which environmental factors influence their activities and functional diversity, measured using Biolog^®^ ECO plates. Our results showed that soil bacterial alpha functional diversity, including substrate richness (number of substrates decomposed) and Shannon diversity, were positively correlated with patch area and number of vascular plant species in the forest floor vegetation layer. However, soil bacterial beta functional diversity (substrate use pattern, CLPP – community level physiological profiles) was primarily driven by patch area and soil physicochemical properties. Our results suggest that the positive effect of patch area (biogeographic effect) on soil bacterial functional diversity may be primarily through stabilisation of environmental conditions, as the amplitude of environmental fluctuations is reduced on larger plots compared to smaller ones. Taken together, our study provides important insights into the relationship between patch area, soil properties, vegetation characteristics, soil bacteria activity, and functional diversity in urban riparian forests, highlighting the importance of considering soil microbes when managing urban ecosystems.

## Introduction

Forest ecosystems are essential for human well-being, providing multiple ecosystem services such as carbon sequestration and biodiversity conservation (Baldrian, 2017; Klimek and Niklińska, 2024). However, their extent and ecological value steadily decrease worldwide due to environmental changes and anthropogenic pressures, including urbanization (Augustynczik et al., 2020; Sabatini et al., 2020). Urban woodlands vary in size, stand age, and management intensity (Pregitzer et al., 2019), resulting in a gradual transition from dense forests to more open, park-like environments (Woźniak et al., 2025). While larger remnants of old-growth forests are preserved in only a few cities (Wang and Yang, 2022), most urban forests consist of small and micro-forests, that are highly fragmented and patchy in their spatial distribution (Picard and Tran, 2021; Ayala-Azcarraga et al., 2023). The spatiotemporal continuity of these urban forest patches is shaped by the expansion dynamic of urban areas (Doroski et al., 2022) and a range of environmental and anthropogenic factors.

Unmanaged urban green spaces are often perceived as having low biological value (Bonthoux et al., 2019), but they have also been shown to represent urban biodiversity hotspots (Hwang and Roscoe, 2017). This is particularly true for floodplains and riverbanks (Hu et al., 2019), which are linked to areas outside of towns through the riverbed as an ecological corridor (Alvey, 2006). Urban riparian forest areas are critical for maintaining ecological connectivity through the river network, acting as a corridor for species movements (Graziano et al., 2022). However, in many cities, extensive sections of rivers are heavily modified or even completely covered by artificial surfaces such as concrete. These alterations may disrupt natural habitats and ecological processes, leading to site fragmentation and loss of biodiversity (Machado and Kim, 2024).

Microbiomes are fundamental components of all ecosystems, playing essential roles in nutrient cycling, organic matter decomposition, and overall ecosystem productivity and health (Gałązka et al., 2022). In the forest ecosystem, soil microbes contribute significantly to energy flow and organic matter cycling, ecosystem biodiversity, and stability (van der Heijden et al., 2008; Baldrian, 2017; Eisenhauer et al., 2017; He et al., 2022). Due to their sensitivity to environmental changes and perturbations, soil microbial parameters (e.g., functional and structural diversity) serve as valuable indicators of ecosystem health (Azarbad et al., 2013, 2016; Fierer et al., 2021; Garg et al., 2024). However, as pointed out by Fierer et al. (2021), interpreting microbial parameters can be challenging due to the complexity and spatiotemporal variability of soil microbiomes. This highlights the importance of context-specific analysis and the integration of microbial indices with soil parameters, such as physicochemical properties, to provide a more comprehensive assessment of ecosystem functions. Urban soils are a specific environment as they suffer from amplified environmental challenges such as increased site fragmentation, soil compaction, temperature, and pollution (Godefroid and Koedam, 2003; McKinney, 2006; Zhang et al., 2020; Nugent and Allison, 2022; Wang M. et al., 2022; Teerlinck et al., 2024). These stressors may alter microbial community structure and function, with recent studies suggesting that urbanization may reduce the complexity and stability of soil microbial networks constructed using amplicon sequencing of bacterial 16S rRNA and fungal ITS genes (Liu et al., 2023) and affect soil microbial enzyme drivers, leading to soil organic carbon loss (Zhang et al., 2024). However, despite their environmental importance, soil microbes associated with urban riparian forests have received less attention (Mgelwa et al., 2019).

Urban green spaces can be highly isolated from each other, which limits the dispersal of organisms (Von Thaden et al., 2021). This may allow urban green spaces to be treated as islands, with their important environmental characteristics such as island size and isolation, which are considered the primary abiotic factors for predicting biodiversity on islands (MacArthur and Wilson, 1967). For macro-organisms, the “island biogeography theory” showed that biodiversity has positive species-area relationships (island-area effect) and negative species-isolation relationships (island-isolation effect) (MacArthur and Wilson, 1967). Some recent reports have shown that these rules can also be applied to micro-organisms (Li et al., 2020; Yang et al., 2021; Raimbaultx et al., 2024). However, the ecological differences between macro-and micro-organisms, particularly different body sizes, indicate that the soil microbial response to site size, site isolation, and site edge length are likely driven by indirect effects (Ewers and Didham, 2007; Xu et al., 2024). These effects may include the vegetation diversity, which is expected to be lower on smaller than larger plots (Olejniczak et al., 2018; Ma et al., 2023); soil properties, which are expected to be more disturbed on smaller than larger plots, i.e., soil texture (Guilland et al., 2018); and the amplitude and severity of changes in environmental fluctuations, such as temperature and moisture, as smaller plots are more susceptible to their effects than larger plots (Yang et al., 2021). Understanding how riparian forest patch area, isolation, and environmental factors shape microbial functional diversity in urban settings will help to inform strategies for preserving biodiversity and microbially-mediated ecosystem services in cities.

The functional diversity of soil microbial communities can be defined as the ability to metabolise different organic compounds (Garcia-Pausas and Paterson, 2011) and can be derived from genetic diversity (Maron et al., 2018). For this study, we chose to use the Biolog^®^ ECO plate method, a phenotypic community-functional approach widely used to assess soil microbial functional diversity (Escalas et al., 2019). This method evaluates the metabolic potential of microbial communities by quantifying their ability to utilize a standardized array of 31 carbon sources. The advantages of the Biolog^®^ approach include its ability to provide direct, community-level functional insights and its suitability for studying fast-growing culturable bacteria. This makes it particularly relevant for understanding functional processes in environments like riparian forest soils.

The present study addressed how the functional diversity of soil bacteria is influenced by site characteristics within willow (*Salix* spp.) dominated forest-like sites in urban area, with limited site connectivity provided by the river network. Our goal was to determine whether soil bacterial community catabolic characteristics are related to riparian forest patch area and/or other environmental characteristics, including vegetation and soil properties. Both vegetation characteristics and soil physical and chemical properties are among the most important factors shaping forest soil microbial communities (He et al., 2022; Khafida et al., 2024). Specifically, we aimed to examine whether the island area influences soil bacteria alpha and beta functional diversity (defined as a number of decomposed substrates and the pattern of substrate use) through affected vegetation characteristics or through soil characteristics or whether these relationships are driven by other mechanisms.

## Materials and methods

### Study sites, vegetation surveys, and soil sampling

Study sites were located in the city of Kraków, the second city in Poland in terms of area (327 km^2^) and number of inhabiting people (0.80 million in the city and 1.5 million in metropolitan area). Kraków is located in the south of Poland: latitude from 19°47′35″E to 20°13′02″ E and longitude from 49°58′04”N to 50°07′32”N. The geological structure varies in different parts of the city, as Kraków lies at the junction of three major geological units: the silesian-cracow Monocline (the Kraków-Częstochowa Upland), the Carpathian foothills (the Carpathian Foothills) and the Outer Carpathians (the Beskids). In the river valleys, the top geological layer consists mainly of Holocene sand and gravel. The climate in the region is temperate with four seasons; the mean annual average temperature (MAAT) is 10.0°C, and the mean annual average precipitation (MAAP) is 700 mm. July is the hottest (19.5°C) and wettest (120 mm) month of the year, while January is the coldest (−2.3°C) and driest (50 mm). The growing season with an average daily temperature above 5°C lasts for 220 days on average. Total forest cover in Kraków is estimated at 4% (Bank Danych Regionalnych, Główny Urząd Statystyczny, 2024), which is one of the lowest value compared to major cities in Poland.

Ten *Salix* spp. dominated, riparian-like forest patches were found in different parts of the city. Geographical coordinates (Supplementary Table S1) and a map of study sites (Supplementary Figure S1) are reported in Supplementary information. Only *Salix* spp. dominated stands were studied to reduce the number of confounding factors, as dominant tree species strongly influence soil microbial characteristics (Chodak et al., 2016). Willow is an important component of temperate riparian areas and provide multiple ecosystem services (Bita-Nicolae, 2023). The ecological continuity of the riparian forest-like vegetation on the study sites lasts up to a few decades, as the riverbanks in the Kraków area have been heavily modified, e.g., by the construction of flood protection systems. The patch area was calculated, with the tree line considered as the boundary of the patch. Despite varying sizes, all patches were ecologically linked because the city’s waterways converge into the Wisła River, which flows through the city center approximately from west to east.

On each patch, a representative 100 m^2^ study plot was delineated in the central part of each site. Vegetation, that is, vascular plants (*N* plant), including trees (*N* tree), shrubs (*N* shrub), and forest floor species (*N* floor), was characterized on each study plot using the Braun-Blanquet method (Braun-Blanquet, 1964). The data on plant cover in the relevés were transformed from the Brown-Blanquet scale into a 0–9 ordinal scale (Van der Maarel, 1979), and the H’_plant_ was calculated on the basis of the Shannon-Wiener general diversity index according to the equation:

$$H\prime_{plant}=-\overset{s}{\underset{i=1}{\sum }}\;p_{i}\left(\log_{10}\;p_{i}\right)$$

where *p_i_* denotes the frequency for the *i*-th species of plant, and *s* is the number of plant species at a particular plot. Within the plant species found, invasive species were separated (*N* invasive), according to Tokarska-Guzik et al. (2012). In each of the 10 study plots, three replicate soil samples were collected diagonally across the plot (two corners and central point), resulting in a total of 30 soil samples (3 samples per plot × 10 plots). The upper 10 cm depth of the soil was collected with a spade. After the transport of soil to the laboratory, the soils were sieved with a 1 cm wide sieve to remove plant residuals, stones, and soil animals and stored at 4°C field moist until further analyses.

### Soil physical and chemical analysis

Soil physical and chemical analyses were carried out on each collected soil sample. The dry weight (DW) of the soil samples was determined by measuring the mass loss (water) after the soil samples had been at 105°C for 24 h. The water holding capacity (WHC), which was the amount of water that a given soil can hold without leaking, was measured using a standard gravimetric method after soil was soaked for 24 h in net-ended plastic pipes immersed in water. The soil pH was measured in air-dried subsamples (2 g) shaken in deionised water (1:10 w:v) for 1 h at 200 rpm. Organic carbon (C) and total nitrogen (N) were analyzed by dry combustion of approximately 5 mg milled soil samples with an elemental analyzer (Vario El III, Elementar Analysen Systeme GmbH). The flow-injection analyzer (FIA compact, MLE) was used to analyze the total P concentration, after wet mineralization of 0.5 g DW of soil subsamples in suprapure 65% HNO_3_ (Merck). To assess the accuracy of the mineralization process, three blank samples and three replicates of standard certified material (CRM025-050, Sandy Loam 8, RT Corp.) were analyzed with the soil samples. The C:N ratio was subsequently calculated to capture the balance between carbon and nitrogen availability. Similarly, the C:P ratio was calculated to provide insight into the interactions between carbon and phosphorus. Particle-size distribution of mineral soil fraction was determined by laser diffraction after a 3 min ultrasound dispersion of the sample in distilled water (Mastersizer 3,000, Malvern Panalytical, United Kingdom) (Gus-Stolarczyk et al., 2022). This analysis provided data on particle size distribution, including percentages of sand, silt, and clay. Each analysis was performed in three subsamples taken from each study plots, and the results are presented as mean values with standard deviations.

### Biolog^®^ ECO plates analysis of soil bacteria

Soil bacteria activity and functional diversity was analyzed using Biolog^®^ Eco plates (Preston-Mafham et al., 2002). The Biolog^®^ Eco plates are 96 well microplates, that contain 3 sets of 31 common carbon sources and employ a tetrazolium redox dye as an indicator of microbial community metabolism of each individual substrate.^1^ The decay of the different substrates in the wells resulted in a change from colourless to purple formazan. The substrates were six compound groups: amines, amino acids, carbohydrates, carboxylic acids, polymers, and others (miscellaneous) (Campbell et al., 1997).

The soil samples (equivalent of 3 g of soil dry mass) were acclimated at 22°C at 60% of their maximal WHC for 4 days and then shaken in 30 mL of 0.9% NaCl at laboratory shaker for 30 min at 200 rpm. The supernatants containing microbes (100 μL) were diluted in 9.9 mL of 0.9% NaCl. Solutions of 100 μL per well were inoculated into the Biolog^®^ Eco plates and the plates were incubated at 20°C in darkness. To prevent contamination, all tools used were sterile. The absorbance in particular wells was measured as light absorbance at 590 nm using a spectrophotometer Tecan with i-control software (Tecan Group Ltd., Männedorf, Switzerland). The first measurement was carried out just after inoculation and then was measured daily for 5 days. The absorbance value for each substrate was corrected by subtracting the value for the control well, which contained no substrate but only the soil suspension. Absorbance changes below 0.06 (spectrometer detection limit) were considered as 0.

Soil bacteria alpha functional diversity was expressed as the number of substrates decayed (R) and by the Shannon diversity index (H’_bact_), which was calculated as:

$$H\prime_{bact}=-\overset{s}{\underset{i=1}{\sum }}\;p_{s}\left(\log_{10}\;p_{s}\right)$$

where *p_i_* is the ratio of the activity on each substrate to the sum of activities on all substrates.

Beta functional diversity of soil bacteria was expressed as patterns of substrate use, commonly called community level physiological profiles (CLPP). The absorbance values for individual substrates were standardized to 1 for each sample to compare relative changes in substrate use pattern.

Soil bacteria activity, that is the overall rate of substrate utilization by microorganisms was expressed by the AUC (Area Under the Curve), which was calculated as follows:

$$AUC=\overset{N}{\underset{i=1}{\sum }}\overset{n-1}{\underset{t=1}{\sum }}\left(\frac{A_{n}+A_{n+1}\;}{2}\right)\times \left(t_{n+1}-t_{n}\right)$$

where *A_n_* and *A*_*n* + 1_ are the absorbance of each well (substrate, n) at two consecutive measurements at two different measurement times for *t_n_* and *t*_*n* + 1_. The final results for each patch were obtained by averaging data from three soil samples from each study plot.

### Statistical analysis

Pearson correlation tests were conducted to examine the relationships between site size, vegetation and bacterial indices and soil properties and to identify independent variables for further analysis. Then, multiple regression analyses were conducted to separately assess the effects of independent factors on the R, H’_bact_, and AUC. The independent factors included in this analysis were patch area, vegetation coverage, number of forest floor plant species, soil pH, soil N and P content, and clay content. These variables were selected based on Pearson correlation coefficients (*r* < 0.6) to represent the wide range of environmental properties and to minimize collinearity. Both backward and forward stepwise selection procedures were performed for each analysis to validate the robustness of the final models. To compare relationships between vegetation diversity and structure and soil bacterial community CLPPs, dissimilarity matrices based on Euclidean distance were calculated, using either the botanical data and Biolog^®^ data. The matrices were then compared using the Mantel test (9,999 permutations) to assess the link between the beta diversity of vegetation and soil bacteria.

Beta diversity refers to the variation in species composition within the community, represented either as a matrix of plant species occurrences or as CLPP data, which reflect substrate utilization patterns by soil bacteria on individual study plots. Next, a partial least squares path modelling (PLS-PM; Sanchez, 2013) was carried out to evaluate the direct and indirect effects of site size, vegetation properties, and soil physicochemical properties. Model was constructed based on weights on standardized manifest variables, and centroid was used for internal estimation. Correlations at significance level 0.001 and goodness of fit indices were calculated for obtained model. Multiple-variable analysis and multiple regression analysis were performed using Statgraphics Centhurion XIX software (StatPoint Technologies Inc., Warrenton VA, United States). Mantel test analysis was performed using PAST 4.10 software (Natural History Museum, University of Oslo, Norway). A partial least squares path modelling (PLS-PM) was carried out with XLSTAT (Lumivero, 2020).

## Results

### Site size and vegetation characteristics

**Figure 1:**
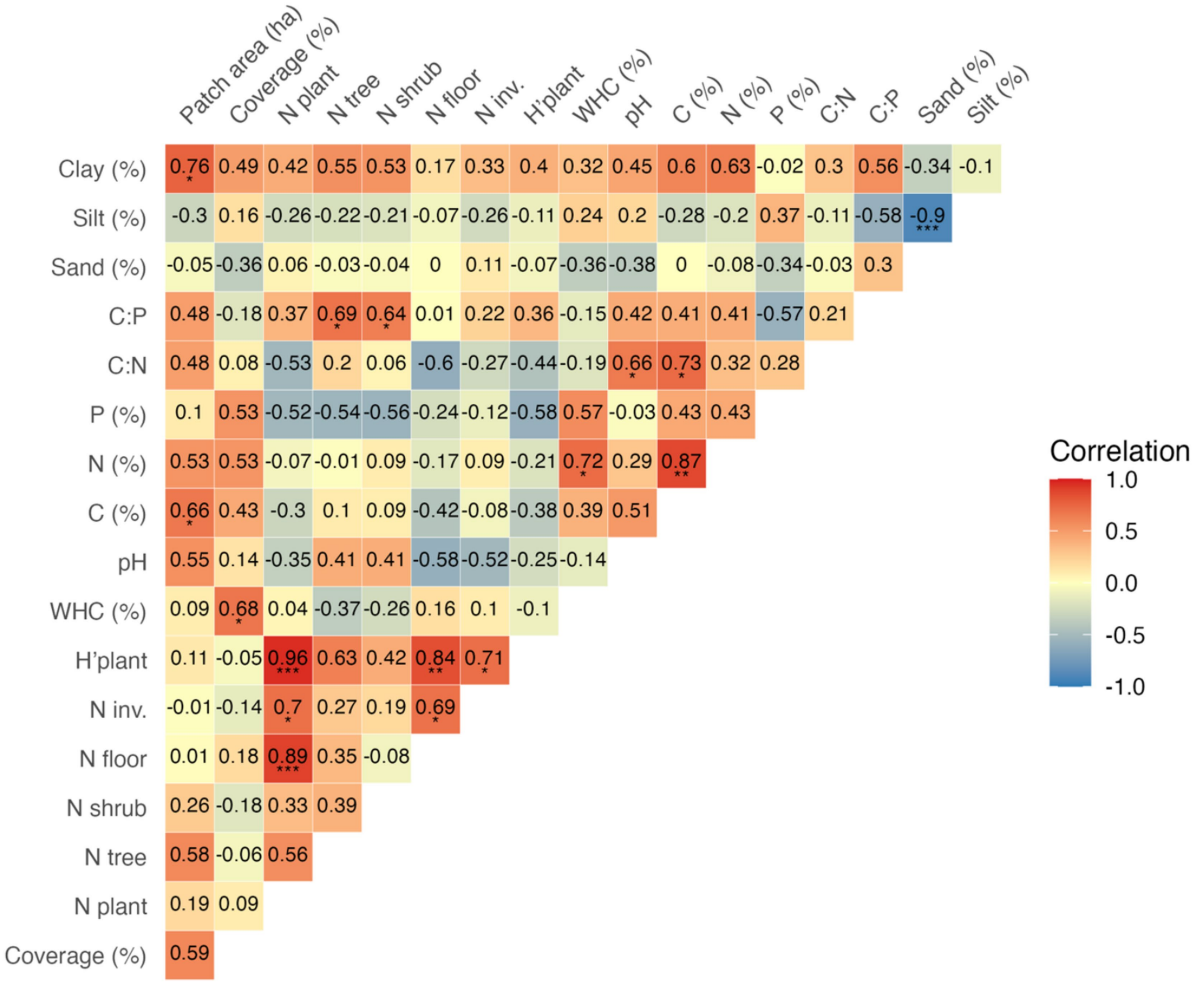
Pearson correlations for riparian-like forest patches data on its area, vegetation properties, and soil properties (*n* = 10). Within each cell, the numeric value indicates correlation strength (scaled from −1 to +1), and asterisks denote significance levels: *, **, and *** for *p* < 0.05, 0.01, and 0.001, respectively. Correlations are displayed in red (positive) and blue (negative); colour saturation denotes the strength of the relationship. Detailed information about soil physicochemical properties, vegetation data, and soil bacterial indices are presented in Tables 1, 2, respectively.

Data on patch area and site characteristics are presented in Table 1. Site size ranged from 0.05 to 1.44 ha. The study plot (100 m^2^) vegetation coverage ranged from 110 to 220%. The number of vascular plant species per plot ranged from 13 to 24, and most of them were forest floor species (61% per plot on average). Dewberry *Rubus caesius*, nettle *Urtica dioica* and avens *Geum urbanum* were the most common forest floor species with the highest plot coverage. Shrubs layer was represented by 2 to 4 species per plot, and the most common were bird cherry *Padus avium* and black elder *Sambucus nigra*. Tree species number per plot varied from 2 to 4, with the predominance of *Salix alba* and *Salix fragilis* (10 and 9 plots, respectively). The vascular plant composition indicated riparian forests of the class *Salicetea purpureae* Moor 1958, but not all plots had the appropriate composition of diagnostic species or were characterized by a low cover of these species (Dzwonko and Loster, 1988; Dzwonko, 2015). A few so-called ancient woodland species were however recorded in some plots: *Aegopodium podagraria*, *Athyrium filix-femina*, *Circaea lutetiana*, *Dryopteris dilatata*, *Dryopteris filix-mas*, *Festuca gigantea* and *Geum urbanum* (Dzwonko, 2015), but most of which had low cover. Number of invasive plant species identified on all plots altogether was 13, with species number per plot ranging from 0 to 5 (15% of plant species per plot on average). *Impatiens parviflora and Impatiens glandulifera* were the most common invasive species (found on 4 and 6 from 10 plots, respectively). Vegetation properties on study plots were highly positively correlated (Figure 1). Plant diversity index (H’_plant_), which ranged from 1.04 to 1.33, was positively influenced by N floor (*r* = 0.84; *p* < 0.001) but also by the number of N invasive species (*r* = 0.71; *p* < 0.05; Figure 1).

**Table 1:** Means, standard deviations, and minimal and maximal values (*n* = 10) for patch area, vegetation data: total coverage (%), total number of plant species (*N* plant), number of tree species (*N* trees), number of shrub species (*N* shrubs) and number of forest floor species (*N* floor), number of invasive species (*N* invasive), plant diversity index (H’_plant_), and soil physicochemical properties.

Variable	Unit	Data set values			
		Mean	SD	Min	Max
Patch area	ha	0.51	0.50	0.05	1.44
Coverage	%	163.5	37.4	110	220
*N* plant	–	17.5	4.0	13	24
*N* tree	–	2.8	0.6	2	4
*N* shrub	–	4.2	1.5	2	7
*N* floor	–	10.5	3.5	6	16
*N* invasive	–	2.6	1.4	0	5
H’_plant_	–	1.2	0.1	1.0	1.3
WHC	% DW	138.8	69.9	40	267
pH	–	7.17	0.63	5.97	8.15
C	% DW	16.39	10.33	1.10	33.60
N	% DW	0.56	0.30	0.06	1.11
P	% DW	0.08	0.03	0.04	0.13
C:N	–	12.6	2.0	10.5	17.2
C:P	–	59.2	26.2	31.8	119.8
Sand	%	13.8	8.92	5	34
Silt	%	74.6	8.44	57	85
Clay	%	11.6	3.86	6	17

### Soil physical and chemical properties

WHC in studied soils ranged from 60 to 127% (Table 1). The pH of the studied soils was neutral to alkaline, with a mean value of 7.17 (± 0.63). The studied soils were characterized by a low content of C, N, and P (4.29, 0.34, and 0.08% on average, respectively). The C:N ratio ranged from 10.5 to 17.2, with a mean value of 12.6 (± 2.0), while the C:P ratio was more variable, ranging from 31.8 to 119.8, with a mean of 59.2 (± 26.2). The soils were mainly composed of silt (mean: 74.6%, ± 8.44), followed by sand (13.8%, ± 8.92) and clay (11.6%, ± 3.86). Patch area was positively correlated only with soil C content (*r* = 0.66; *p* < 0.05) and clay percentage (*r* = 0.76, *p* < 0.001), as shown in Figure 1. Soil C content was, in turn, positively correlated with soil N content (*r* = 0.87; *p* < 0.01). Plot coverage was positively correlated only with soil WHC (*r* = 0.68; *p* < 0.001). C:N ratio was positively correlated with soil pH (*r* = 0.66; *p* < 0.001; Figure 1).

### Soil bacteria activity and functional diversity

**Figure 2:**
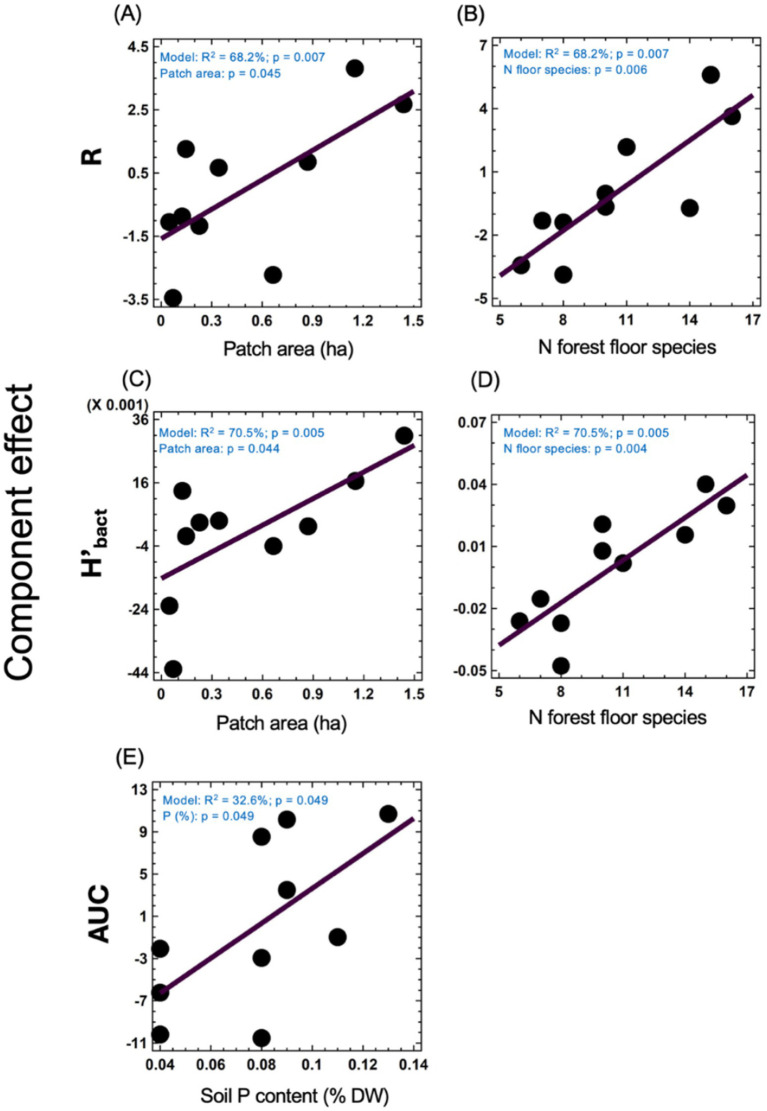
Component effects from multiple regression analysis on the relationships between environmental factors and bacterial parameters. Effect of patch area **(A)** and the number of forest floor plant species **(B)** on R (number of substrates decomposed by bacteria). Influence of patch area **(C)** and the number of forest floor plant species **(D)** on H’bact (Shannon diversity index of bacterial functional diversity). Effect of soil phosphorus (P) content on AUC (overall bacterial activity) **(E)**. Dots are individual patches (*n* = 10). Model parameters, including adjusted R^2^ and overall model *p*-values, are indicated in blue text within each panel. Individual *p*-values for the factors are also provided.

**Figure 3:**
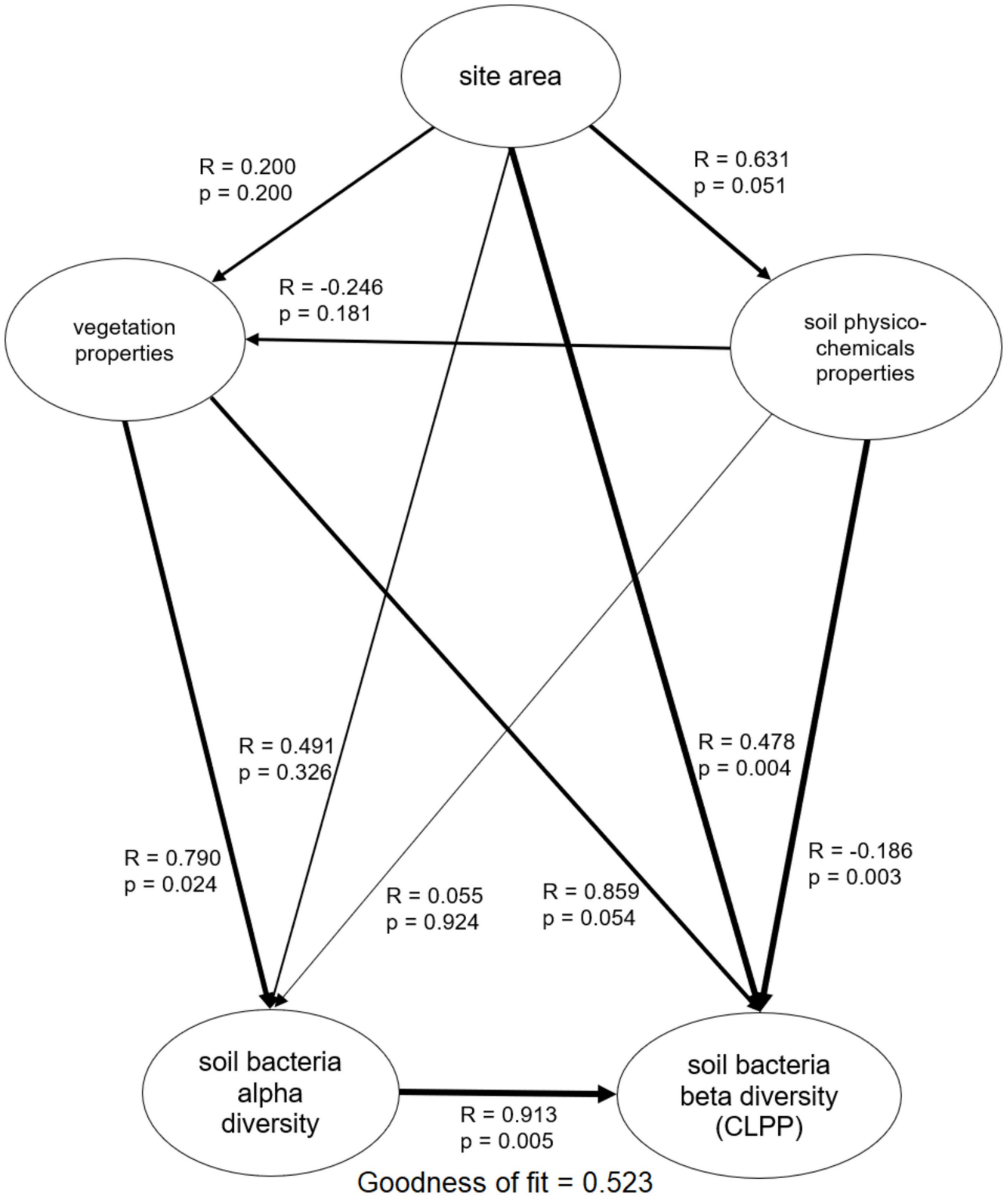
Partial least squares path models (PLS-PM) displaying the direct and indirect effects of the interaction of site size and vegetation effect on soil bacteria functional diversity (alpha diversity and beta diversity). Arrows width presents correlation strength, values by the individual arrows denote correlation (R) value and significance of the effect (*p* value).

AUC ranged from 45.8 to 67.0 (Table 2; Supplementary Figure S2). R ranged from 18 to 28, meaning that 76% of substrates on Biolog^®^ ECO plates was decomposed on average, indicating relatively high functional diversity in studied soils. H’_bact_ ranged from 1.20 to 1.30. Carboxylic acids, carbohydrates and amino acids were among the most used substrate groups, for each soil representing above 70% of the response (Table 2; Supplementary Figure S2). The multiple regression analysis was performed to study the effect of patch area and plot properties on soil bacteria activity and alpha functional diversity indices (Figure 2). The output of these analyses indicated that models were significant for R, H’_bact_, and AUC. For R, the model explained 68.2% of the variance (*p* = 0.007), which was positively dependent on the patch area (*p* = 0.045) (Figure 2A) and the number of forest floor plant species (*p* = 0.006) (Figure 2B). For H’_bact_, the model was significant (*p* = 0.005) and explained 70.5% of the variance. H’_bact_ showed a significant positive correlation with the site size (*p* = 0.044) (Figure 2C) and the number of forest floor plant species (*p* = 0.004) (Figure 2D). For AUC, the model explained 32.6% of the variance (*p* = 0.049), where AUC values were positively dependent only on soil P content (*p* = 0.049) (Figure 2E). Mantel test results revealed that plant community beta diversity was not correlated with bacteria functional beta diversity (CLPP) (*p* = 0.458, *R* = 0.017). The PLS-PM confirmed these results (Figure 3; Supplementary Figure S3), where soil bacterial alpha functional diversity indices (R, H’_bact_) were mainly determined by vegetation characteristics. In turn, soil bacteria beta functional diversity (CLPP) was primarily driven by site area and soil physicochemical properties.

**Table 2:** Means, standard deviations and minimal and maximal values (*n* = 10) for soil bacteria alpha functional diversity, that is R (number of substrates used) and H’_bact_ (Shannon diversity), and soil bacteria activity (AUC) and structure of substrate groups use (relative % of use).

Variable	Unit	Data set values			
		Mean	SD	Min	Max
R	–	23.5	3.4	18.8	28.3
H’_bact_	–	1.26	0.06	1.20	1.30
AUC	–	56.3	8.0	45.8	67.0
Amines	%	7.7	1.6	5.2	10.6
Amino acids	%	19.5	1.9	15.8	21.2
Carbohydrates	%	24.7	2.6	20.5	27.4
Carboxylic acids	%	27.8	2.6	23.8	31.3
Miscellaneous	%	8.0	1.6	5.7	11.4
Polymers	%	12.3	1.8	9.3	15.1

## Discussion

Soil microorganisms in urban areas provide essential ecosystem services such as those involved in nutrient cycling, erosion control, and regulation of climate change stressors (Metzler et al., 2024). Therefore, it is highly important to better understand how human-based activities influence microbial diversity and functions in urban ecosystems. Indeed, the conservation of soil biological diversity is an extremely important task in a rapidly changing world under human activity, and site fragmentation is one of the most challenging problems. Our study provides important insights into the relationship between patch area, soil properties, vegetation characteristics, soil bacterial activity, and functional diversity in urban riparian forest patches. Using Biolog^®^ ECO plates, we showed that larger riparian forest patches exhibited higher values of the R measure, reflecting the percentage of substrates decomposed, as well as increased bacterial alpha functional diversity (H’_bact_). These findings suggest that larger riparian forest patches in urban environments may provide improved habitat conditions, which in turn support higher functional diversity of soil bacteria.

It is important to note that temperate riparian forests are regarded as a threatened or endangered habitat in Europe (Czortek et al., 2020; Przepióra and Ciach, 2022). This is particularly true for the vegetation, an important component that contributes to the biotic part of this unique ecosystem. On average, about 30 species of vascular plants per plot (100 m^2^ of phytosociological relevance) are reported in riparian forests in Poland, and in well preserved regions even 50 species per plot (Macicka and Wilczyńska, 1988; Matuszkiewicz, 2008). In our study, the mean number of plant species per 100 m^2^ plot was much lower and amounted to 17.1 (± 3.8). Similar results were obtained by Stefańska-Krzaczek (2013), who identified 13.7 (± 4.9) species in a single phytosociological relevé in riparian forest remnants in Wrocław, the third largest city (in terms of population) in Poland. Plant species richness and diversity are considered to be one of the most important factors shaping soil microbial communities (Toju and Sato, 2018). On the other hand, certain soil microbial groups may increase ecosystem stability, especially under stressful conditions, by facilitating nutrient acquisition for plant communities.

We used Biolog^®^ tests to study the functional (catabolic) diversity of soil bacteria. However, the limitations of such an approach need to be taken into account while interpreting our results. For instance, this method allows the study of only those fractions of bacteria that can be extracted and cultured. In addition, direct comparisons of results obtained in different laboratories must be carefully preceded by a review of the details of the laboratory analysis, such as the degree of dilution of the inoculum (bacterial suspension) applied to the wells of the plate, which can strongly influence the final results. In general, data on the functional diversity of soil bacteria under *Salix* spp. stands are scarce and mostly related to post-mining sites (Kaneda et al., 2019). Soil bacterial activity and functional diversity in *Salix* spp. dominated riverbanks in urban areas were comparable with data obtained for other types of forests in Poland, using the same laboratory protocol. In particular, Klimek et al. (2016) found that in different nearly undisturbed temperate forest types, the mean values for AUC, R and H’_bact_ in soil A horizon were 39.0, 24.4, and 1.10, respectively. Wasak et al. (2019), in their study conducted in a forest mountainous area near Kraków, found that averaged values for AUC, R, and H’_bact_ were 31.1, 19.5, and 1.21, respectively. The values of soil bacterial indices measured in a current study were slightly higher than those obtained in near-natural temperate forests in Poland. This could be due to various environmental factors, such as soil pH. In natural temperate forests, which are mostly characterized by acidic pH, soil bacteria may be outcompeted by soil fungi. Soil pH was neutral to alkaline in the riparian soils, and higher soil pH may favour bacteria over fungi (Wang and Kuzyakov, 2024). Moreover, urban forest soil is becoming alkaline under rapid urbanization (Zhang et al., 2023). Soil bacteria AUC was, however, not related to soil pH, but only to soil P content. Phosphorus is an essential nutrient not only for plants, but also for microorganisms (Wang Z. et al., 2022). Although it contributes to diffuse pollution and eutrophication, riparian soils are highly effective at sorbing readily soluble forms of phosphorus (Frątczak et al., 2019). Phosphorus supports rapid plant biomass production, which in turn promotes soil microbial performance (Chen and Xiao, 2023). Willow is known for its fast growth rate (Koczorski et al., 2022). Phosphorus fertilisation has been shown to increase crop yield in willow short rotation coppice for biomass production (Kuzovkina et al., 2018), and similar effects can be expected in riparian areas.

Soil bacteria alpha functional diversity indices, both R and H’_bact_, increased with the number of forest floor plant species, which represents the majority of plant diversity in temperate forests (Gilliam, 2007). Vegetation influences a wide range of soil microbial parameters (Tilman et al., 1997). For example, plant species-rich communities produce more biomass and more chemically diverse litter and root exudates (rhizodeposition), which essentially contribute to higher soil C content, as we showed in this study. Higher plant species richness also promotes plant-microbes interactions, which can, in turn, lead to the establishment of more diverse microbial communities (Prober et al., 2015). Indeed, soil bacteria are effective in utilizing simple organic compounds, delivered by plant roots, which are generally more available in nutrient-rich habitats such as a rhizosphere (Wang and Kuzyakov, 2024). The chemical groups of carbon substrates most used by bacteria on Biolog^®^ plates were carboxylic acids, carbohydrates and amino acids. These substrate chemical groups are particularly essential components of root exudates, which are known to support the growth and development of soil bacterial communities (Wang and Kuzyakov, 2024), and therefore differentiation between the experimental treatments with these substrate groups was often reported (Furtak et al., 2020). Although we did not observe a significant relationship between soil bacterial functional beta diversity (CLPP pattern) and vegetation diversity and composition as showed by Mantel test, our findings align with previous studies indicating that vegetation diversity has a greater impact on fungal communities than on bacterial communities. For instance, using amplicon sequencing of bacterial 16S and fungal internal transcribed spacer (ITS), Štursová et al. (2016) investigated bacterial and fungal communities and diversity in a regenerating temperate mountain forest in the Bohemian Forest, Central Europe. Their results revealed that fungal communities exhibited significantly higher beta diversity (variation among communities) than bacterial communities, with fungi being more strongly influenced by vegetation. In our study, another potential factor contributing to the lack of a clear relationship between soil bacterial beta functional diversification and vegetation beta diversity could be due to a relatively high proportion of invasive plant species. Previous studies have shown that cities are hotspots for alien species, with over 35˗40% of them have been recorded in cities (Pyšek, 1998; Clemants and Moore, 2003). Native plant species are generally more abundant than non-native species (Knapp et al., 2008), but in some places non-native plant species may dominate native ones. River valleys are particularly vulnerable to invasion by alien species (Pielech, 2021). In disturbed anthropogenic habitats, the number of alien plant species tends to correlate with the total number of plant species (Siwek et al., 2024). Invasive plant species can affect plant communities and soil physicochemical and microbiological properties, all of which can affect soil carbon dynamics (Raheem et al., 2024).

Multiple regression analysis confirmed that soil bacterial alpha functional diversity indices, both R and H’_bact_, increased with patch area, but to a greater extent from vegetation diversity, that is, forest floor species number. However, PLS-PM indicated a negligible direct effect of patch area on alpha functional diversity indices. In contrast, patch area had a significant effect on the beta diversity of soil bacteria (CLPP), which seems to confirm that the island effect on soil bacteria is driven by habitat heterogeneity. This habitat heterogeneity likely operates at the microscale within soil samples, as indicated by the significant correlation between patch area with soil clay and C contents. The role of soil texture in shaping microbial diversity is well-supported by previous studies. For instance, Biesgen et al. (2020) showed that clay content modulates bacterial community composition by increasing aggregate stability, which fosters the development of distinct microbial communities in microaggregates. In our study, the positive correlation between patch area and clay content may explain the observed influence of patch area on bacterial beta diversity, as larger patches tend to accumulate finer particles, creating heterogeneous microhabitats that enhance bacterial community differentiation. Moreover, clay content was observed to correlate with soil moisture due to the occurrence of micropores and menisci that generate capillary forces (Richert et al., 2009; Bicharanloo et al., 2022).

A larger patch area may support ecosystem functions better than a smaller one through multiple mechanisms. One such mechanism is the mitigation of direct anthropogenic disturbances such as soil compaction and soil pollution. This was demonstrated by Kostrakiewicz-Gierałt et al. (2022) in their study of urban forests and parks in Kraków, where larger patches tended to have greater resilience and functionality in the face of human-induced stressors. However, urban riparian forest, including small ones, are difficult to access due to dense vegetation cover, and direct human activity is limited. Another possible mechanism is the greater resistance of larger areas to water shortages, a factor that may be particularly relevant in riparian areas. In general, urban hydrology is drastically altered compared to agricultural and natural areas (Pickett et al., 2001). The high proportion of impervious surfaces in urban areas, combined with the vegetation structure characterized by reduced larger tree cover in urban green spaces, leads to increased rainwater runoff. Coupled with changes in the structure of precipitation under global climate change, i.e., a higher proportion of torrential rainfall, the increased runoff in urban areas changes the morphology of urban streams, which become deeply incised in their floodplains (Soboyejo et al., 2025). This hydrological modification can isolate remnant riparian vegetation from the water table, compounding the challenges posed by reduced groundwater availability during droughts. During periods of drought (reduced rainfall), water deficiency may limit soil microbial activity, both directly through reduced soil water content but also by limiting the morphological and physiological traits of plants, including a reduction in fine root biomass and their carbon contribution to the soil below ground (Hinko-Najera et al., 2015). This hypothesis can also be supported by the positive correlation observed in this study between soil water holding capacity and vegetation coverage. Furthermore, the positive correlation between plot area and soil clay content suggests that larger plots may experience less soil erosion, as clay particles, being the smallest and most easily transported by water, are more likely to be retained in these areas compared to smaller plots.

It has been shown that soil bacteria are more sensitive to drought than fungi (Azarbad et al., 2018, 2020; de Vries et al., 2018). Li et al. (2020) investigated the island biogeography of soil bacteria and fungi (via amplicon sequencing) and revealed that the diversity of soil bacteria alpha diversity is strongly influenced by soil moisture. Their study demonstrated that smaller islands, characterized by lower soil moisture and greater edge effects, exhibited reduced bacterial diversity compared to larger islands. However, it is important to note that soil moisture is a highly viable soil property that could be influenced by temporal weather conditions during sampling rather than a consistent indicator of soil property. Moving forward, future studies should focus on the potential role of larger patch areas in urban riparian forests in supporting soil health and ecosystem functions, particularly in relation to water availability and resistance to drought. Such studies should include a more detailed investigation of soil microbiomes, including bacteria and fungi, based on amplicon and metagenomic sequencing assays. Integrating microbial sequencing approaches with CLPP data helps to link phenotypic functional diversity with the underlying genetic and taxonomic drivers. Additionally, the “island effect” on soil microbial diversity and functionality may vary depending on the type of island, whether of natural or anthropogenic origin. This highlights the importance of comparative studies across different island types.

## Conclusion

In conclusion, our study confirms that bigger riparian forest patches in urban areas support for higher functional diversity of soil bacteria. We showed that alpha functional diversity of soil bacteria was mainly driven by vegetation characteristics, whereas beta functional diversity was primarily by site size and soil physicochemical characteristics. Conservation of green urban areas is receiving increasing attention, as they can support important parts of the biodiversity of the geographical region, up to the national scale (Basak et al., 2021). It is particularly important to mitigate the urban heat island effect in the face of global climate change (Teerlinck et al., 2024), as it can severely impact residents in densely populated urban areas. There is a need to prevent the loss of biodiversity in disturbed landscapes, in particular in urban areas (Hansen et al., 2005). Our study may serve as an argument for preserving at least a part of urban green spaces in their natural state. It will be important to validate our findings by investigating the impact of the island effect and climate change on soil microbial community structure and functioning in urban riparian areas, with a focus on the conservation of urban green spaces to support biodiversity and mitigate the effects of urbanization.

## Data Availability

The original contributions presented in the study are included in the article/Supplementary material, further inquiries can be directed to the corresponding author.

## Footnotes

^1^ http://www.biolog.com
