# Supplementary material for: Size matters: larger fragments of riparian forest in urban areas support functional diversity of soil bacteria more than smaller ones
Source: Front Microbiol. 2025 Feb 26;16:1517545. doi: 10.3389/fmicb.2025.1517545 (PMC11897014; doi:10.3389/fmicb.2025.1517545)
Supplement: Supplementary file 1 [file Data_Sheet_1.docx]

**Suplementary Material**

Article: Size matters: larger fragments of riparian forest in urban areas support functional diversity of soil bacteria more than smaller ones

Gabriela Koster ^1^, Małgorzata Jaźwa ^2^, Sebastian Wojciech Przemieniecki ^3^, Łukasz Musielok ^4^, Hamed Azarbad ^5^*, Beata Klimek ^1^

Table S1. Study plots location (neighboring street) with riverbank/stream names if applied (Wisła tributares mostly) and geographical coordinates.

|  | Location | Watercourse | GPS location | |
| --- | --- | --- | --- | --- |
|  |  |  | Latitude (N) | Longitude (E) |
| 1 | Kosocicka | Malinowka | 50.003966 | 20.010076 |
| 2 | Dzikiej Rozy | Wilga | 49.995655 | 19.927951 |
| 3 | Zolla | Serafa | 50.005218 | 20.036066 |
| 4 | Czarnochowicka | Serafa | 50.008399 | 20.060072 |
| 5 | Wankowicza | Dłubnia | 50.07833 | 20.056258 |
| 6 | Podbipięty | Dłubnia | 50.052428 | 20.06462 |
| 7 | Zielone Wzgorze | Prądnik | 50.107448 | 19.933406 |
| 8 | Norymberska | old riverbed | 50.035855 | 19.898009 |
| 9 | Odmętowa | old riverbed | 50.061471 | 20.042482 |
| 10 | Tuchowska | stream without name | 50.002714 | 19.969026 |


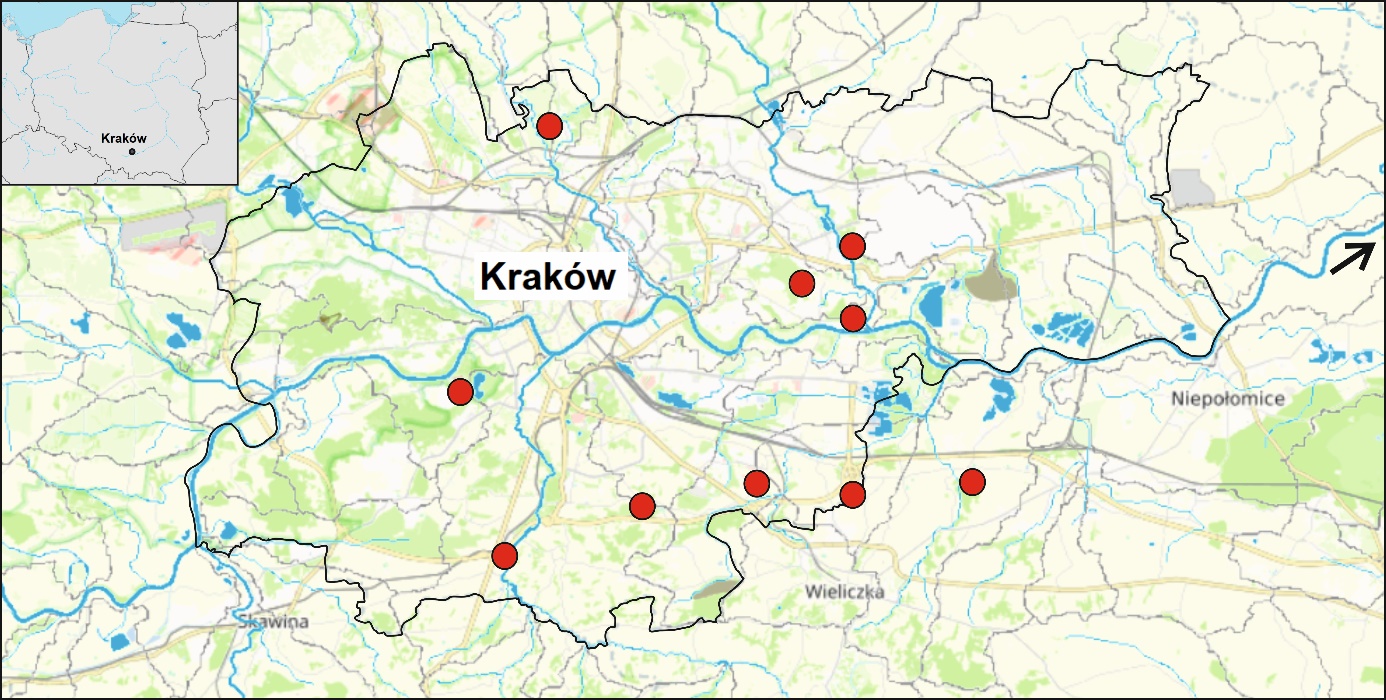


**Figure S1**. Localisation of the study area in a wider geographical context and distribution of the study plots (red dots) on the map of Kraków (the administrative boundaries of the city are marked with a black line). Green colour indicates green areas of different types.

Blue colour indicates rivers and water bodies; the thinnest blue lines are tertiary tributaries. The black arrow on the edge of the map indicates the direction of the main river Wisła. The river network has been presented according to the EU-Hydro database (EEA, 2019).


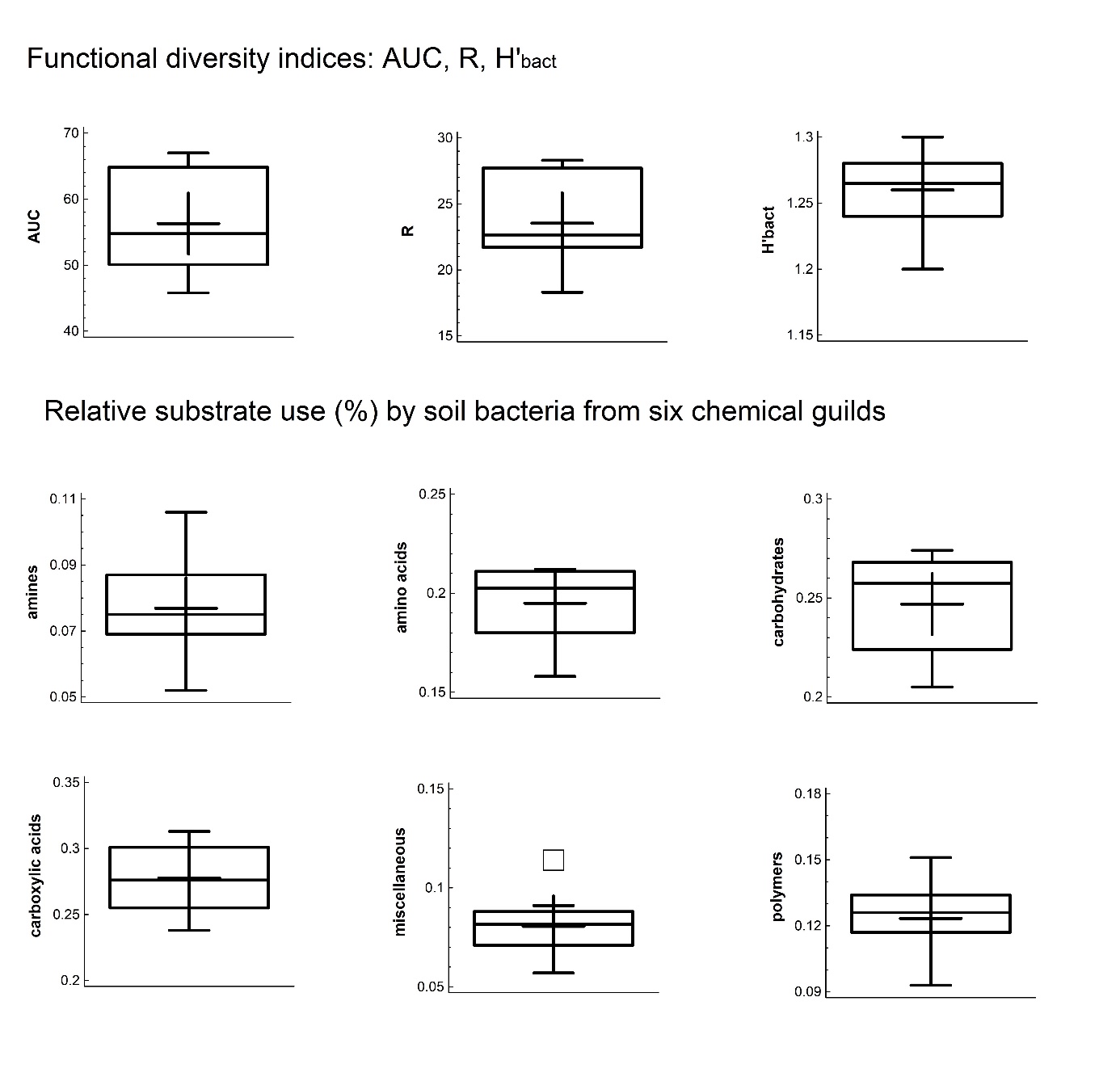


Figure S2. Box plots presenting data distribution for variables measured. Each box is drawn extending from the lower quartile of the sample to the upper quartile, that is the interval covered by the middle 50% of the data values when sorted from smallest to largest. A vertical line is drawn at the median and a plus sign is placed at the location of the sample mean. Whiskers are drawn from the edges of the box to the largest and smallest data values, unless there are values unusually far away from the box (which Tukey calls outside points). Outside points, which are points more than 1.5 times the interquartile range (box width) above or below the box, are indicated by point symbols.


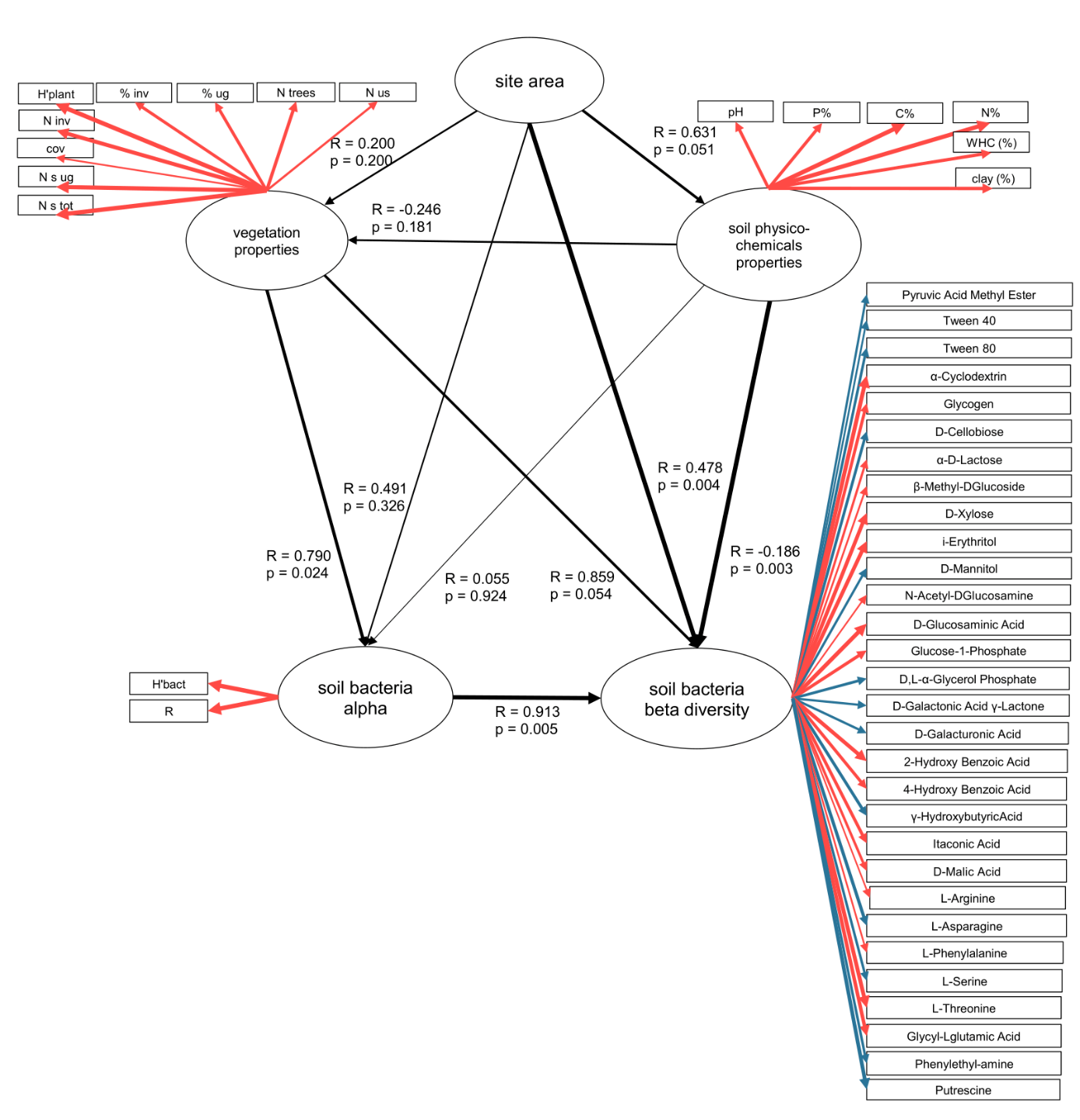


Figure S3. Partial least squares path models (PLS-PM) displaying the direct and indirect effects of the interaction of site size and vegetation effect on soil bacteria functional diversity (alfa diversity and beta diversity). Black arrows width presents correlation strength, values by the individual arrows denote correlation (R) value and significance of the effect (p value). Red arrows indicate positive loading factors, blue arrows indicate negative loading factors.

Reference:

EEA, 2019. Copernicus Land Monitoring Service Reference Data: EU-Hydro. In. European Environment Agency (EEA).
